# VetTag: improving automated veterinary diagnosis coding via large-scale language modeling

**DOI:** 10.1038/s41746-019-0113-1

**Published:** 2019-05-08

**Authors:** Yuhui Zhang, Allen Nie, Ashley Zehnder, Rodney L. Page, James Zou

**Affiliations:** 1 Department of Computer Science and Technology, Tsinghua University, Beijing, China; 2 Department of Biomedical Data Science, Stanford University, Stanford, CA 94305 USA; 3 Department of Clinical Sciences, Colorado State University, Fort Collins, CO 80523 USA; 4 Chan-Zuckerberg Biohub, San Francisco, CA 94158 USA

## Abstract

Unlike human medical records, most of the veterinary records are free text without standard diagnosis coding. The lack of systematic coding is a major barrier to the growing interest in leveraging veterinary records for public health and translational research. Recent machine learning effort is limited to predicting 42 top-level diagnosis categories from veterinary notes. Here we develop a large-scale algorithm to automatically predict all 4577 standard veterinary diagnosis codes from free text. We train our algorithm on a curated dataset of over 100 K expert labeled veterinary notes and over one million unlabeled notes. Our algorithm is based on the adapted Transformer architecture and we demonstrate that large-scale language modeling on the unlabeled notes via pretraining and as an auxiliary objective during supervised learning greatly improves performance. We systematically evaluate the performance of the model and several baselines in challenging settings where algorithms trained on one hospital are evaluated in a different hospital with substantial domain shift. In addition, we show that hierarchical training can address severe data imbalances for fine-grained diagnosis with a few training cases, and we provide interpretation for what is learned by the deep network. Our algorithm addresses an important challenge in veterinary medicine, and our model and experiments add insights into the power of unsupervised learning for clinical natural language processing.

## Introduction

Large-scale electronic health records (EHR) can be a powerful resource for patient care and research. There have been many exciting efforts applying machine learning to human medical records—e.g. predicting in-hospital mortality, 30-day unplanned readmission, and prolonged length of stay^1,2^ —with the goal of assisting medical professionals. In comparison to the human EHR, there has been little machine learning (ML) work on veterinary EHR, which faces several unique challenges. While it is standard practice for clinicians to enter standardized diagnosis and billing codes for human EHR, almost all veterinary clinics lack resources to annotate their patient notes with standard diagnosis coding. Veterinary records can be extremely valuable for research and public health—60–70% of all emerging diagnoses are transmitted from animals to humans. Beyond that, companion animals have been increasingly used to study naturally occurring diseases as they share similar environments to humans and are often more representative disease models compared with induced mouse models, which frequently do not accurately recapitulate diseases in humans. While cancer is a leading area of cross-species translational studies,^3^ other diseases such as genetic neuromuscular disorder,^4^ osteoarthritis^5^ and diabetes^6^ are being studied in companion animals as well. The lack of standard diagnosis coding on veterinary records is a major bottleneck for public health monitoring and these cross-species translational studies.^7^

Inferring diseases and diagnoses from free text such as diagnostic reports and clinical notes has been actively studied in clinical natural language processing (NLP).^8^ However, most of these works are designed for human EHR. They are often trained and evaluated on clinical notes gathered from the same hospital as well. Veterinary notes have different styles and vocabulary, and its diagnosis codes use a terminology framework different from humans. Therefore an automated veterinary coding algorithm is needed. Moreover, due to the lack of general coding practice in the veterinary clinics, algorithms can only be trained on coded notes collected from a handful of training hospitals, but need to maintain high performance when they are applied to notes from a diverse set of clinics across the country. Clinical notes from different clinics can differ substantially in its writing style, making automated coding a challenging task.

Processing free text such as diagnostic reports and clinical notes, as well as generating structured information understandable by human have been a central focus of clinical natural language processing.^8^ Most of the previous research has focused on the human healthcare systems, assisting a wide range of clinical operations such as adenoma detection, assisting billing code assignment,^9^ and discovering novel phenotypes and diagnoses using unsupervised learning method on a large set of multimodal data.^10^

Previous work has also focused on searching for effective architectures for the automated coding of human diagnoses, from applying the long short-term memory networks (LSTM),^11^ multi-level hierarchical text processing models,^12^ to memory condensing networks.^13^ Rajkomar et al. have also proposed using deep learning models to predict a wide range of quantities in electronic medical record.^1^ Learning text representation that generalizes across domains is the goal of many recent papers. These promising results share the same approach: pretrain the model on a large unlabeled text corpus using unsupervised learning objectives. Such unsupervised pretraining allows the model to achieve state-of-the-art results on many tasks such as question answering, named entity recognition, and commonsense reasoning.^14,15^

Veterinary clinical notes, due to the lack of infrastructure and third-party payer system, are almost entirely uncoded, making it challenging to analyze the record for diagnosis prevalence, outcome studies, and drug adverse effects. A recent method, DeepTag, takes the first step toward addressing this challenge.^16^ DeepTag predicts 42 top-level diagnosis codes from veterinary clinical notes by training a deep learning model on the Colorado State University Veterinary (CSU) dataset. Although the training dataset is large, DeepTag suffers from significant performance drop when it is deployed to another set of notes collected from a private practice. This new work differs from DeepTag as we augment supervised training with a form of unsupervised learning – language modeling to read through millions of unlabeled notes provided by another hospital. Such unsupervised training is a promising new approach to boost the power of many clinical NLP methods on both human and veterinary data.

SNOMED-CT codes, similar to other structured diagnostic codes assigned to clinical notes, are designed to form a hierarchy. DeepTag predicts whether a given note fits in with a subset of the 42 broad diagnosis codes, corresponding to the highest level of SNOMED-CT hierarchy. It does not predict specific diagnoses. The challenge with directly predicting each fine-grained diagnosis code is that there are thousands of diagnoses and many of them are rare in the training set. Perotte et al. had proposed a training method for support vector machine (SVM) to leverage the hierarchy and alleviate the problem of low recall on very rare label classes.^17^ In this work, we extend this hierarchical training method to neural network classifiers and apply it to veterinary diagnosis coding to predict 4577 SNOMED-CT codes with high performance.

We develop a large-scale algorithm, VetTag, that automatically predicts thousands of fine-grained veterinary diagnosis codes from free-form veterinary notes. Our algorithm is trained on a curated dataset of over 100 K expert labeled veterinary notes and over one million unlabeled notes. We adapt the new state-of-the-art Transformer model proposed by Vaswani et al.,^18^ and demonstrate that large-scale language modeling on the unlabeled notes substantially improves coding accuracy. We systematically evaluate the model performance in challenging settings where VetTag trained on one hospital is evaluated in a different hospital with substantial domain shift. We use hierarchical training to alleviate data imbalances and demonstrate such training scheme substantially benefit rare diagnoses. In addition, we provide interpretation for what is learned by the deep network. VetTag addresses an important application in healthcare and our experiments add insights into the power of unsupervised learning for clinical natural language processing.

## Results

### Problem definition

**Fig. 1:**
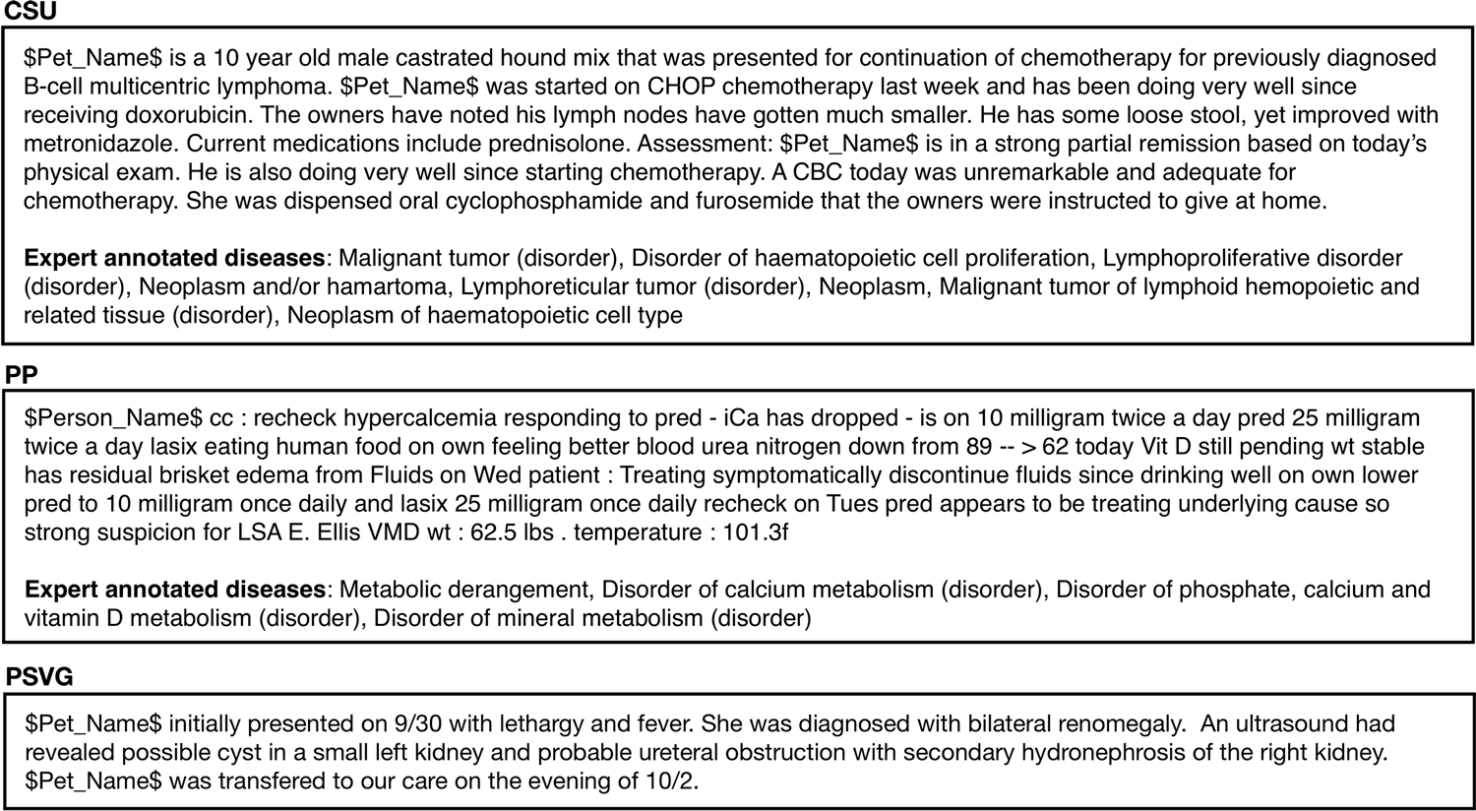
Example clinical notes from the Colorado State University (CSU), a private practice clinic (PP) and a private specialty veterinary group (PSVG) datasets. CSU and PP are expert labeled and PSVG is unlabeled

VetTag takes a free-text clinical note as input and infers a set of clinical diagnoses from the note. The inferred diagnosis is in the form of SNOMED-CT codes and each note can be associated with multiple codes if the patient has several diagnoses. Figure 1 provides examples of veterinary notes from Colorado State University (CSU), a private practice clinic in Northern California (PP), and a large private specialty veterinary group (PSVG) that we use to train and evaluate our coding algorithms. CSU and PP notes are expert labeled with the relevant SNOMED-CT codes, and PSVG is unlabeled.

**Fig. 2:**
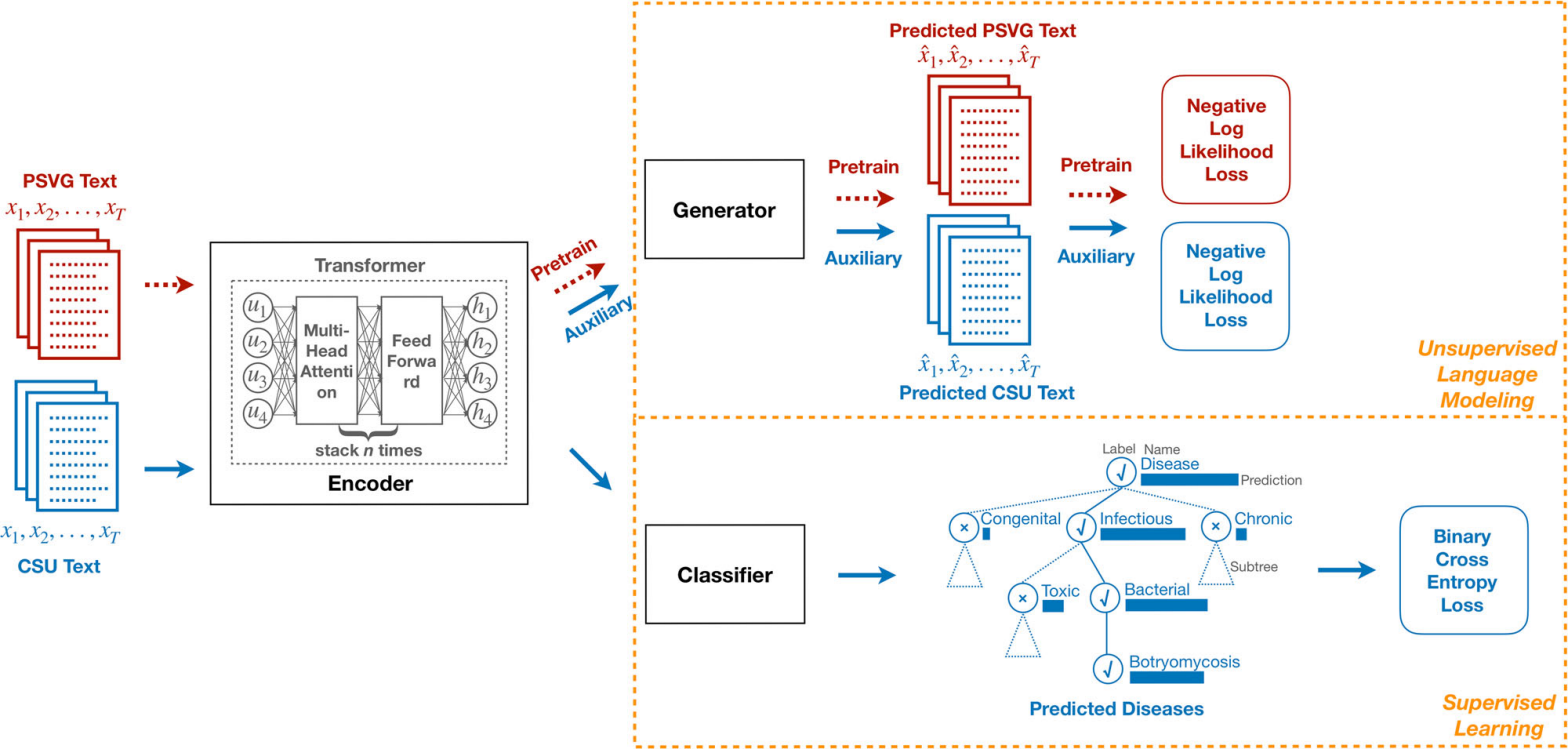
Our proposed model architecture for automated diagnosis coding. Two tasks are shown: unsupervised language modeling (top) and supervised learning (bottom). The dashed red arrows represent the pretraining process on the unlabeled PSVG data, and the solid blue arrows represent the fine-tuning process on the labeled CSU data. Additional test is done on the PP data (not shown)

VetTag is trained in two stages: unsupervised learning and then supervised learning. During the unsupervised learning stage, we train VetTag on 1,019,747 unlabeled veterinary clinical notes from a large private specialty veterinary group that operates multiple specialty clinics (PSVG) to simply predict the next word conditioned on all previous words. The goal of this unsupervised learning is to “familiarize” VetTag with medical concepts and writing, so that it can more efficiently learn from the labeled data. During the supervised learning stage, we train VetTag on 112,557 labeled veterinary notes from the Colorado State University of Veterinary Medicine and Biomedical Sciences (CSU). VetTag adapts the Transformer architecture as the encoder^18^ to generate a contextualized vector representation for the input text, and predicts the diagnosis using the vector. Figure 2 provides a schematic overview of VetTag and details of the model are provided in Supplementary Materials.

**Fig. 3:**
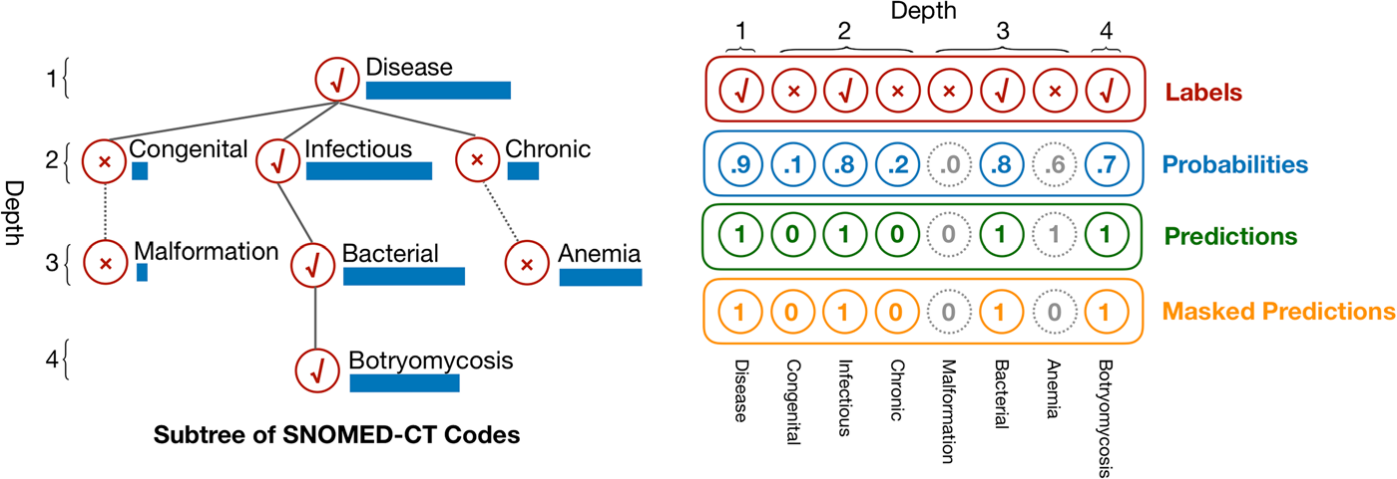
Example of hierarchical training. We show a 4-layer subtree of SNOMED-CT Codes in the left part and its vector representation in the right part. Each node is labeled with diagnosis name, depth, true diagnosis label (marked as check or cross) and VetTag’s predicted probability (shown as the horizontal bar). During training, we only consider the binary cross entropy loss for nodes whose parent diagnosis is present in the expert label—each node is linked with a solid line in the tree. *Malformation* and *Anemia* are not taken into consideration, i.e. they are masked, because their parents *Congenital* and *Chronic* are not present in the expert labels. These masked nodes are linked with dotted edges. The masked predictions are not used to update the model during training

VetTag aims to predict whether each of the 4577 SNOMED-CT diagnosis codes applies to the clinical note. A major challenge here is the large number of potential diagnoses and the fact that many of the codes are rare in the dataset. We leverage the hierarchical structure of SNOMED-CT codes to improve VetTag training. In the SNOMED-CT hierarchy, the top level codes (i.e. depth 1 and 2 starting from the top) correspond to broad diagnosis categories, while the lower level codes are increasingly more fine-grained diagnoses. Instead of predicting all of the codes in parallel, we use a hierarchical prediction approach where VetTag first predicts the top level codes and then sequentially predicts on a child diagnosis when its parent diagnosis is predicted to be present. This approach enables VetTag to leverage the relations between the diagnosis. Figure 3 provides an example of the hierarchical training and more details are in the Methods Section.

We evaluate VetTag’s performance on two datasets. One contains a set of holdout non-overlapping 5628 notes randomly selected from the CSU dataset. The second is an external validation dataset that we collected from a different commercial hospital PP that contains 586 documents. CSU dataset contains notes collected from a tertiary referral academic hospital—the writing tends to be more polished and longer. PP and PSVG notes are collected from primary and secondary referral hospitals, where the notes are shorter and written with expediency using more abbreviations. We provide a comparison of these datasets in Supplementary Figs. 1 and 2.

### Performance evaluation

We systematically compare the performance of VetTag on both CSU and PP test data with commonly used non-deep learning algorithms (MetaMap) and standard deep learning algorithms based on convolutional neural networks (CNN),^19^ long short-term memory networks (LSTM),^20^ as well as recent variations including the state-of-the-art model on MIMIC — an open data of ICU medical records^21^ (CAML),^22^ bidirectional LSTM (BLSTM) and pretrained LSTM encoder with auxiliary language modeling objective (LSTM + AP). As none of these algorithms have been applied to this veterinary coding task previously, we trained our own implementations for the purpose of this comparison. Table 1 reports the performance of all of the algorithms. Each algorithm is evaluated based on prediction precision (the fraction of predicted diagnoses that match the expert diagnoses), recall (the fraction of the expert diagnoses that are successfully retrieved), *F*_1_ (the harmonic mean of precision and recall), and exact match (EM, the fraction of notes where the algorithm’s predicted diagnoses exactly match the expert diagnoses). Since there are 4577 possible diagnoses, getting an exact match is quite challenging. VetTag achieves the best performance across all of the metrics. The improvement over other algorithms is especially notable for the PP test data, demonstrating that VetTag is more robust to variations across different hospitals and clinics.

**Table 1:** Evaluation of trained classifiers on the CSU test data and PP data

Model	CSU				PP (Cross-hospital)			
	F1	Prec	Rec	EM	F1	Prec	Rec	EM
**VetTag**	**66.2**	**72.1**	**63.1**	**26.2**	**48.6**	**54.9**	**47.7**	**9.2**
MetaMap (SVM)	56.8	56.4	57.7	8.9	32.7	35.7	37.3	0.0
MetaMap (MLP)	50.8	55.2	47.5	13.8	21.6	27.3	20.2	0.3
CNN	62.7	75.6	55.8	20.2	33.1	42.0	30.7	1.9
CAML	62.6	74.1	56.2	17.8	37.7	54.7	32.0	3.1
LSTM	60.1	72.4	53.4	22.3	30.3	49.9	24.1	7.5
BLSTM	60.2	70.6	54.5	20.2	35.5	50.9	30.2	4.4
LSTM + AP	45.3	63.8	38.7	12.5	31.3	48.9	26.3	2.2
Transformer	38.2	55.3	32.2	13.9	22.9	34.8	22.3	2.2
Transformer + W	44.6	61.6	38.0	14.8	29.0	45.6	25.1	1.7
Transformer + P	63.3	76.6	56.1	22.8	30.1	56.4	24.3	6.5
Transformer + A	63.5	72.2	58.3	20.9	41.2	51.6	37.3	5.5
Transformer + AP	64.8	74.4	59.8	20.3	45.0	53.1	42.7	7.0

EM is the fraction of cases where the set of diagnoses predicted by the model *exactly matches* the expert labels. The classifiers are trained on a subset of CSU. Notation: CNN, LSTM and Transformer are our base models; BLSTM is bidirectional LSTM; CAML is the state-of-the-art model on MIMIC, an open data of ICU medical records. +W uses Word2Vec trained on PSVG to initialize; +P uses language modeling objective trained on PSVG to initialize; +A uses language modeling objective on CSU in addition to classification objective on CSU; Hierarchical uses hierarchical loss during training process; VetTag trains a transformer with auxiliary objective (+A), pretraining (+P) and hierarchical loss

As discussed above, VetTag combines the recently developed Transformer model^18^ with an auxiliary language modeling objective (A), pretrained encoder (P) and the SNOMED hierarchical loss. In order to evaluate the contribution of each of these components, we also systematically quantify the model’s performance when each of these three components is removed. Using only a subset of these components leads to strictly worse performance on both CSU and PP test data, indicating that they are all required to produce the optimal results.

DeepTag is the previous state-of-the-art algorithm for automated veterinary diagnosis.^16^ DeepTag is a bidirectional LSTM trained to predict 42 top-level SNOMED diagnoses, and can not be directly applied to predict the 4577 fine-grained codes we are interested in here. Therefore we can not compare it directly in Table 1; the DeepTag architecture is the most similar to BLSTM. In order to head-to-head compare VetTag with DeepTag, we restrict predictions to 41 top-level diagnoses except for clinical finding (the spurious category) and report its results in Supplementary Table 1 and Supplementary Fig. 3. Note that since VetTag is optimized for all 4577 diagnoses and DeepTag is optimized for only 42 diagnoses, this comparison is favorable for DeepTag. Despite this, VetTag and DeepTag achieve similar accuracy on the CSU data, with VetTag having higher EM score, and VetTag is substantially better on the PP test data.

### Performance analysis

#### Language model helps Transformer

Training a system on multiple tasks with shared encoding can often improve the model’s performance on all tasks, as different tasks serve as implicit regularization to prevent the model from overfitting to a particular task.^23^ In our experiment, we compare the performance of our system by adding language modeling objective as an auxiliary task during the classification task (Transformer + A vs. Transformer in Table 1). Adding the language modeling as an auxiliary task improves Transformer CSU test set as well as the cross-hospital PP evaluation set. We also combine the language modeling pretraining as well as the auxiliary task during the classification task and observe a substantially better performance on the overall model compared to the baseline model with either approach alone (Transformer + AP vs. Transformer + A or Transformer + P in Table 1).

#### Hierarchical training improves performance

Diagnosis codes at a greater depth in the SNOMED hierarchy tend to be more specific, and thus fewer positive cases can be found for it. In the traditional multilabel classification setting, rare diagnoses will have significantly more negative labels than positive labels, encouraging the classifier to always output a negative label. We use hierarchical training to address this imbalance problem. We report the performance comparison by depth in Table 2. We observe more significant improvement as depth increases when we use hierarchical training compared to the same model with the standard non-hierarchical loss (Transformer + AP). In Table 3, we give samples of the representative diagnoses and VetTag’s performance at the first five depth levels.

**Table 2:** Comparison of tagging performance by depth with/without hierarchical training

Dataset	Depth	#Diagnosis	#Case	Without Hierarchical				With Hierarchical			
				F1	Prec	Rec	EM	F1	Prec	Rec	EM
CSU	1	56	91109	76.2	81.8	74.1	51.3	76.6	83.8	72.9	52.7
	2	299	90880	73.3	79.1	70.0	35.9	73.8	78.3	71.3	38.9
	3	632	89856	66.9	75.9	61.1	31.0	68.1	72.9	65.1	33.3
	4	1086	85783	62.6	73.3	56.7	33.7	63.9	69.7	60.9	33.8
	5	1298	70242	55.6	68.4	49.8	45.8	57.7	65.2	54.2	44.1
	6	804	46250	45.2	62.7	39.7	68.2	49.4	59.2	45.7	65.5
	7	283	12994	37.9	54.7	31.1	90.2	45.3	56.1	43.3	89.7
	8	66	2918	19.7	41.9	14.4	97.4	31.7	44.1	31.5	97.4
PP	1	56	497	57.8	61.8	57.2	26.8	57.7	67.6	54.9	25.3
	2	299	495	52.4	56.3	52.0	13.8	55.5	58.7	56.5	15.0
	3	632	489	46.0	54.5	42.4	14.2	50.2	55.6	49.8	11.9
	4	1086	462	43.4	54.7	39.3	16.9	46.9	54.1	45.2	14.5
	5	1298	389	28.3	38.7	26.9	25.8	33.4	42.2	31.3	24.6
	6	804	216	16.1	28.6	17.7	58.9	22.9	28.7	21.6	54.3
	7	283	68	10.9	10.3	13.0	86.5	14.9	24.3	14.5	86.3
	8	66	9	18.2	50.0	11.1	95.1	0.0	0.0	0.0	97.8

Data are more unbalanced as depth increases, and thus we observe more significant improvements by hierarchical training

**Table 3:** Label performance by depth

Depth	Diagnosis	CSU					PP (Cross-hospital)				
		#	F1	Prec	Rec	EM	#	F1	Prec	Rec	EM
1	Disease by body site	84832	91	90	92	87	461	83	84	82	74
	Inflammatory disorder	25271	72	77	68	89	193	64	73	57	79
	Infectious disease	11304	60	70	52	93	88	42	68	31	87
2	Disorder of body systems	79365	90	88	91	85	459	83	85	81	74
	Disorder of soft tissue	36237	75	78	73	85	205	65	57	76	72
	Disease of trunk	35398	78	77	79	86	147	56	53	59	77
3	Malignant tumor	28058	91	93	89	96	19	52	36	90	95
	Inflam. of specific body systems	23911	72	71	73	88	190	66	66	67	78
	Inflam. of specific body organs	22531	72	71	73	89	170	66	65	68	80
4	Disease of abdomen	20215	73	71	75	90	90	44	46	42	84
	Disease of digestive organ	19136	68	70	65	90	177	55	62	50	76
	Disease of digestive tract	17997	71	75	68	92	184	60	69	53	78
5	Disease of upper digestive tract	11316	65	69	61	94	154	57	65	51	80
	Disease of gastrointestinal tract	9265	70	74	67	96	33	29	29	30	92
	Disorder of anterior eye segment	7638	80	77	83	97	38	58	56	61	94

We sample three of the five most frequent diagnoses from each layer and report its performance for each depth. Diagnoses are more specific as layer goes deeper

#### VetTag achieves good performance across species

Our CSU training and test data contain a broad range of animal species, with canine being the dominant species (over 75% of the dataset). In the PP test data, we observe that canine make up around 70% of the cases and a larger portion of feline. In Table 4, we break down the test performance of VetTag for each species. Overall, VetTag achieves the highest *F*_1_ on the canine cases, and slightly lower performance for feline and equine cases. We provide statistics on the number of notes per species for both CSU and PP data in Supplementary Fig. 2.

**Table 4:** VetTag performance stratified by species

Species	CSU					PP (Cross-hospital)				
	#	F1	Prec	Rec	EM	#	F1	Prec	Rec	EM
Canine	4351	67.2	73.3	63.9	24.5	425	49.7	56.9	48.2	7.8
Feline	607	59.8	64.1	58.8	23.4	149	43.9	46.8	45.4	12.8
Equine	549	61.3	65.1	60.3	39.7	0	0.0	0.0	0.0	0.0
Bovine	60	47.8	55.0	46.1	40.0	0	0.0	0.0	0.0	0.0
Caprine	21	39.5	36.8	45.0	38.1	0	0.0	0.0	0.0	0.0
Porcine	26	63.6	77.2	57.6	38.5	1	31.2	31.2	31.2	0.0
Ovine	8	54.7	52.0	60.9	50.0	0	0.0	0.0	0.0	0.0
Other Mammals	6	56.9	53.8	62.9	33.3	10	53.1	54.2	54.3	10.0

#### MetaMap fails to extract discriminative information

We investigate the effectiveness of traditional feature extraction techniques provided by MetaMap, which is a popular method in medical NLP for extracting medically relevant keywords from text.^24^ We apply MetaMap directly to each veterinary note to extract a bag-of-keywords. Then we use either Support Vector Machine (SVM) with the linear kernel or Multilayer Perceptron (MLP) as the classification algorithm from scikit-learn.^25^ We treat these as our baseline and report the result in Table 1. We find that MetaMap features are not very discriminative at identifying diagnoses in the veterinary medicine domain, and its performance is worse than our various baselines on both the CSU and PP test data.

#### Pretrained language model outperforms Word2Vec

Perplexity is a common metric to evaluate the quality of a language model; lower the perplexity, higher the quality.^26^ Our Transformer model achieves a test perplexity of 15.6 on the PSVG dataset, which is substantially better than the 20.7 perplexity achieved by LSTM on the same data. We also note that compared to the state-of-the-art perplexity achieved on other corpora such as Wall Street Journal or Wikipedia, 47.69 and 40.68 respectively,^27^ the perplexity we obtained is much lower, signaling that the clinical notes are much more structured than other sources of written text. In the experiments reported in Table 1, we also find that language modeling as pretraining is sufficient for models to learn useful word embeddings—model with +*P* outperforms model using Word2Vec embedding trained on PSVG (+*W*) on both CSU and the cross-hospital dataset PP.

### Interpreting how VetTag works

**Fig. 4:**
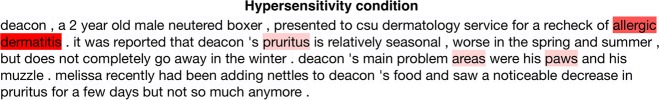
Example of text interpretation from the CSU dataset. Words positively contributing to the predicted label are highlighted in red by the gradient map

In order to better understand how VetTag predicts diagnosis codes from clinical notes, we implement a simple saliency-based interpretation method for VetTag. The saliency of each word quantifies how much that word influences VetTag’s predictions, and it is computed as the gradient of the predicted probability with respect to the input word. We show an example of the keywords highlighted by saliency scores in Fig. 4—the higher the saliency score, the darker the color and the more influential is the word to VetTag’s prediction. We report the top ten most salient words for ten top-level diagnosis codes that overlap DeepTag’s diagnosis codes in Table 5. The full list of salient words for all the top-level diagnosis codes is provided in Supplementary Table 2. More precisely, for each diagnosis category, we compute the medical words that are the most likely to be salient—i.e. with saliency score ≥0.2, a score chosen to select on average 11 words per note—and report these words. Words captured by the model have high quality and agree with medical domain knowledge. Most words captured by the model is in the expert-curated dictionary from the MetaMap. Moreover, we notice that the model is capable of capturing abbreviations (i.e., ‘kcs’—keratoconjunctivitis sicca), combinations (i.e., ‘immune-mediated’) and rare professional terms (i.e., ‘cryptorchid’) that MetaMap fails to extract.

**Table 5:** Most salient words for VetTag

Diagnosis (SNOMED-CT code)	Extracted keywords
Traumatic AND/OR non-traumatic injury	fracture, wound, laceration, due, assessment, trauma, this, bandage, time, owner
Visual system disorder	eye, ophthalmology, surgery, eyelid, assessment, sicca, time, uveitis, diagnosed, this
Hypersensitivity condition	dermatitis, allergic, therapy, atopic, otitis, pruritus, ears, assessment, allergies, dermatology
Metabolic disease	diabetes, nph, hypercalcemia, glargine, vetsulin, weeks, home, insulin, amlodipine, dose
Anemia	pancytopenia, anemia, visit, hemolytic, persistent, steroids, hypertension, neoplasia, exam, thickening
Disorder of immune function	eosinophilic, then, problem, todays, hypocalcemia, cornea, dose, skin, alt, weeks
Disorder of endocrine system	methimazole, thyroid, weeks, levothyroxine, carcinoma, mass, hyperadrenocorticism, assessment, diabetes, diagnosed
Disorder of connective tissue	osteosarcoma, assessment, ligament, surgery, carboplatin, disease, dysplasia, rupture, cruciate, fracture
Poisoning	ingestion, assessment, toxicity, chocolate, vomiting, charcoal, not, maya, chance, activated
Congenital disease	dysplasia, hip, bilateral, assessment, testicle, right, cerebellar, service, surgery, echo

We select ten representative diagnosis categories. For each diagnosis, we show the top 10 words in the MetaMap medical dictionary that the model most strongly associates with the phenotype. Words are sorted in decreasing order by its frequency in the CSU test set

## Discussion

Processing veterinary clinical notes and generating structured information has a tremendous impact on the ecosystem of veterinary clinical data science. In this study, we extended the previous work in two important directions.^16^ First, we propose a language model framework to leverage a massive amount of unlabeled clinical notes, demonstrating that this type of unsupervised learning is crucial in improving the performance and robustness of the diagnosis coding model. Second, we build a system to predict 4577 SNOMED codes—DeepTag was also able to predict 42 top-level diagnosis codes by comparison—by leveraging the hierarchy amongst the SNOMED codes so that the model only predicts the child diagnosis when all of its parents are present. We demonstrate that this hierarchical training is significantly better than the standard multi-label prediction scheme especially for rare diagnosis categories which previously suffered from low recall. We show that training with diagnosis hierarchy not only improves performance on the original task, but also improves the robustness of VetTag when it is applied to data from a different clinic.

We analyze the impact of depth (specificity) of a diagnosis to the performance of the model. Clinical note coders are instructed to apply lower-level, more specific codes as much as they can. Many labeled codes correspond to very specific diagnoses, and simply predicting top-level diagnosis is not sufficient in practice. As specificity and depth increase, the number of potential diagnoses also increases and the number of relevant cases decreases. With hierarchical training, we find a substantial improvement for the more specific diagnosis.

We additionally provide a saliency method to explain VetTag by visualizing the words in the clinical note that most significantly influences VetTag’s prediction. The most salient words for VetTag agree well with the clinically meaningful terms. Moreover, VetTag saliency map identifies words such as acronyms and combinations beyond what the standard MetaMap vocabulary. Highlighting such salient in clinical notes can help human curators to label documents more quickly and provide rationalization over the VetTag’s decision process.

As we make meaningful progress toward a more robust automated coding system for veterinary medicine, we note that there is still a significant drop in performance when applied to text from a different hospital. The significant improvement over the baseline methods as well as the ability to infer a wide range of diagnosis codes gives us cautious optimism to apply this tool to label veterinary clinical notes and conduct analyses. However, due to the inherent bias from our training data, some important diagnoses such as *neoplasm and/or hamartoma* are over-represented, resulting in lower precision when applied in the cross-hospital setting. We can partially mitigate this effect by adjusting the decision threshold of the binary classifier, but further research needs to be conducted on learning both over-represented diagnoses and under-represented diagnoses in this setting. An important step of future work will be to fully study the cross-hospital performance of our algorithm by collaborating with other veterinary academic institutions, and conduct pilot studies that integrate VetTag into the veterinary IT infrastructure. There could be potential values to mapping SNOMED labels to a restricted subset of codes that are currently used in clinical practices. We focused on SNOMED because it is commonly used and we believe similar model as VetTag can be used to predict other codes with potentially even better accuracy. This is a good direction of further work.

## Methods

### Datasets

We use three datasets in our experiments (Table 6). Three examples are sampled from each dataset and shown in Fig. 1.

**Table 6:** Descriptive statistics of the three datasets

	CSU (Labeled)	PP (Labeled)	PSVG (Unlabeled)
# of notes	112,557	586	1,019,747
# of training set	101,301(90%)	0(0%)	917,665(90%)
# of validation set	5,628(5%)	0(0%)	51,103(5%)
# of test set	5,628(5%)	586(100%)	50,979(5%)
Avg # of words	368	253	72

#### Labeled data 1: Colorado State University (CSU)

We use a curated set of 112,557 veterinary notes from the Colorado State University College of Veterinary Medicine and Biomedical Sciences. Each note is labeled with a set of SNOMED-CT codes by veterinarians at Colorado State. Colorado State is a tertiary referral center with an active and nationally recognized cancer center. We find 4577 total SNOMED codes present in the CSU labeled dataset. These represent the relatively more common diagnosis and we focus on predicting these codes.

#### Labeled data 2: private practice (PP)

We also use a smaller set of 586 discharge summaries curated from a commercial veterinary practice located in Northern California. Two veterinary experts applied SNOMED-CT codes to these records. Records with coding discrepancies were reviewed by both coders to reach a consensus on each record. This dataset is drastically different from the CSU dataset. PP notes are written often in an informal style, evidenced by their shorter length and usage of abbreviations. The PP data also has a different diagnosis distribution compared to a specialized academic cancer center CSU.

#### Unlabeled data: private specialty veterinary group (PSVG)

We obtained a large set of over one million unlabeled notes from a large private specialty veterinary group that operates multiple veterinary clinics. This is a set of raw clinical notes without any codes applied to them.

#### Data Processing

We filter out all non-ASCII characters in our documents, convert all letters to lower case, and then tokenize with NLTK.^28^ We apply the standard BPE (Byte Pair Encoding)^29^ algorithm to address the out-of-vocabulary problem, and to speed up the language modeling training. BPE uses a vocabulary size of 50 K, and out-of-vocabulary words are encoded as subword units. We randomly split the CSU and PSVG dataset into training, validation and test set for supervised learning and unsupervised learning.

#### SNOMED-CT Codes

SNOMED-CT is a comprehensive clinical health terminology managed by the International Health Terminology Standards Development Organization.^30^ Annotations are applied from the SNOMED-CT veterinary extension (SNOMED-CT VET), which is a veterinary extension of the International SNOMED-CT edition. In this work, we try to predict disease-level SNOMED-CT codes.

ICD-9/ICD-10 billing codes are the results of complex interactions between the patient, care-provider, potentially third-party coders and insurance policies, all of which could introduce systematic bias in what codes are assigned.^31^ In order to reduce potential biases, the SNOMED-CT VET codes in our dataset are assigned by veterinary school students using standardized procedures to facilitate cohort identification and record retrieval for clinical science.

Disease-level SNOMED-CT codes are organized as a directed acyclic graph. However, there are only a small number of nodes with more than two ancestors. By applying the breadth-first search algorithm from the root node, the general disease in SNOMED-CT codes, we can get the shortest path from the root node to any specific diagnosis node. For each node, we only reserve the shortest path from the root node. The directed acyclic graph is transformed to a tree after processing. For each node, depth represents for the distance from the root node to the current node, and branch represents the number of children of the current node. We show statistics of processed disease-level SNOMED-CT codes in Table 7.

**Table 7:** Descriptive statistics of the disease-level SNOMED-CT codes

	Mean	Std	Min	Max	Median
Depth (Distance from root)	5.0	1.5	0	11	5
Branch (# of children)	1.9	8.9	0	891	0

### Algorithm development and analysis

We build the base of our model using the multi-layer Transformer architecture similar to the setup in Radford et al.^15^ We concisely summarize the VetTag algorithm here and more details are provided in Supplementary Materials.

We model automated coding as a multi-label classification problem. Given a note, we want to predict whether the note is positive (i.e. supports the diagnosis) of each diagnosis label *y* in a predefined set of diagnoses ${\cal{Y}}$. For the *i*-th diagnosis, we want to predict whether the binary diagnosis label *y*_*i*_ is 0 or 1. Here each label corresponds to a SNOMED-CT diagnosis code. Our proposed model architecture is shown in Fig. 2. Three tasks are shown: unsupervised learning, supervised learning and hierarchical training. We describe these three tasks in the following section and details are provided in Supplementary Materials.

#### Unsupervised Learning

We build a generative model over text for unsupervised learning, also referred to as a language model. Text sequence is an ordered list of tokens. Therefore, we can build an autoregressive model to estimate the joint probability of the entire text sequence *X*: *p*(*X*) = *p*(*x*_1_, …, *x*_*T*_), where *x*_*t*_ represents the *t*-th token in the sequence of length *T*. In an ordered sequence, we can factorize it as $p(X) = \mathop {\prod}\limits_{t = 1}^T p (x_t|x_1,...,x_{t - 1})$. Concretely, we estimate the token distribution of *x*_*t*_ by using the contextualized representation vector $h_t \in {\Bbb R}^d$ provided by our encoder: *h*_*t*_ = Encoder(*h*_1_, …, *h*_*t*−1_), where *d* is latent dimensions of the model. We optimize over the negative log-likelihood of the distribution $- \log p(X) = - \mathop {\sum}\limits_{t = 1}^T {\log } p(x_t|x_1,...,x_{t - 1})$.

In our model, we examine the effect of language modeling on two encoder architectures: Transformer and the long short-term memory (LSTM). We use this objective in two parts of our system: (1) *pretrain* encoder’s parameters; (2) serve as an *auxiliary task* during training of the classifier.

#### Supervised Learning

We get a summary representation vector $c \in {\Bbb R}^d$ for the entire sequence from the encoder. We then use a fully connected layer to down project it and calculate the probability of whether *j*-th diagnosis should be predicted: $p(y_j) = \sigma (w_j^Tc + b_j)$, where $w_j \in {\Bbb R}^d$ and $b_j \in {\Bbb R}$ are the weight and bias for the classifier of *j*-th diagnosis, and *σ* is the sigmoid function: *σ*(*x*) = 1/(1 + *e*^−*x*^). We compute the binary cross entropy loss $L({\cal{C}})$ across *m* labels: $L({\cal{C}}) = - \frac{1}{m}\mathop {\sum}\limits_{j = 1}^m {y_j} \log p(y_j) + (1 - y_j)\log (1 - p(y_j))$, where binary label *y*_*j*_ ∈ {0, 1} indicates whether *j*-th diagnosis is true in the expert label.

Finally, we use a mixture of two losses $L_{{\mathrm{total}}} = L({\cal{C}}) - \lambda \ast \log p(X)$ and use hyperparameter *λ* = 0.5 to set the strength of the auxiliary loss when we use language modeling as an *auxiliary task* in our classification training.

#### Hierarchical Training

There are less training cases for a more specific diagnosis. The severe data imbalance for certain diagnosis makes classifier tend not to predict these diagnoses. We alleviate the problem by utilizing hierarchy in SNOMED-CD codes. Instead of predicting each diagnosis individually, we predict diagnosis from top to bottom, and we call it hierarchical training. We show an example in Fig. 3.

For training, we update the classifier using its prediction of a diagnosis only when all the ancestors of this diagnosis are true in the expert label. In practice, we ignore the binary cross entropy loss of the diagnosis if any ancestor of this diagnosis is not true in the expert label.

For prediction, we only predict that the diagnosis is true when all the ancestors of the diagnosis are predicted as true. In practice, we predict the diagnosis as false if any of the ancestors of the diagnosis has been predicted to be false.

## Supplementary information

Supplementary

## Data Availability

The data were made available to Stanford for the current study, and are not publicly available. The data are available from the authors upon reasonable request and with permission of the veterinary centers.

## Supplementary information

**Supplementary information** accompanies the paper on the *npj Digital Medicine* website (10.1038/s41746-019-0113-1).

## Code availability

VetTag is freely available at https://github.com/yuhui-zh15/VetTag.
