# Supplementary material for: VetTag: improving automated veterinary diagnosis coding via large-scale language modeling
Source: NPJ Digit Med. 2019 May 8;2:35. doi: 10.1038/s41746-019-0113-1 (PMC6550141; doi:10.1038/s41746-019-0113-1)
Supplement: Supplementary file 1 — Supplementary [file 41746_2019_113_MOESM1_ESM.pdf]

Supplementary Figures

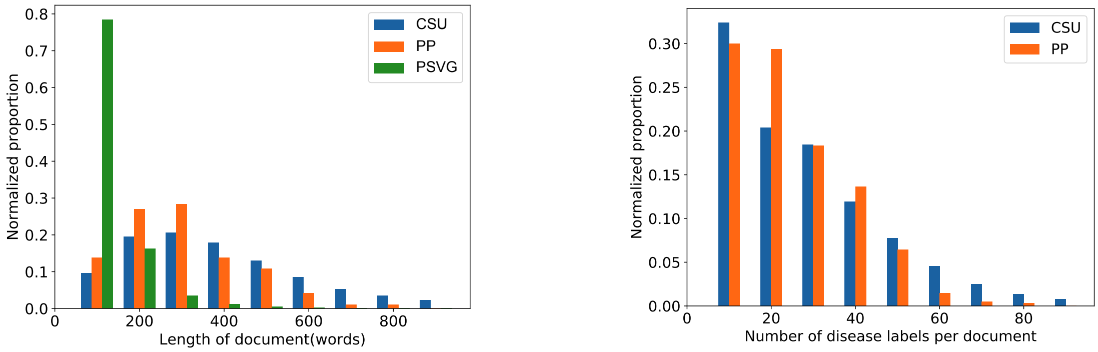

Supplementary Figure 1: Document length distribution (left) and label number distribution (right) in CSU, PP and PSVG dataset.

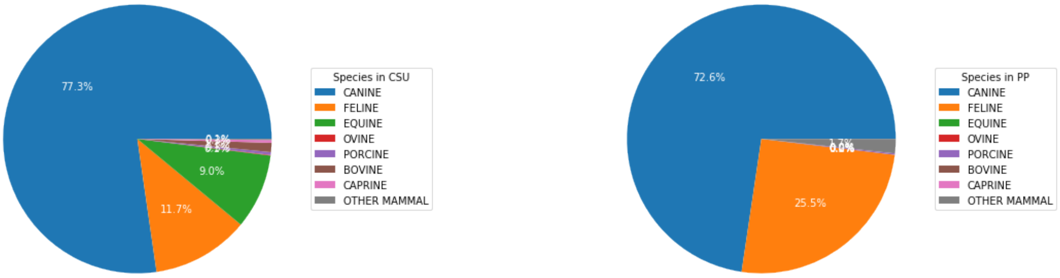

Supplementary Figure 2: Species distribution in CSU dataset (left) and PP dataset (right).

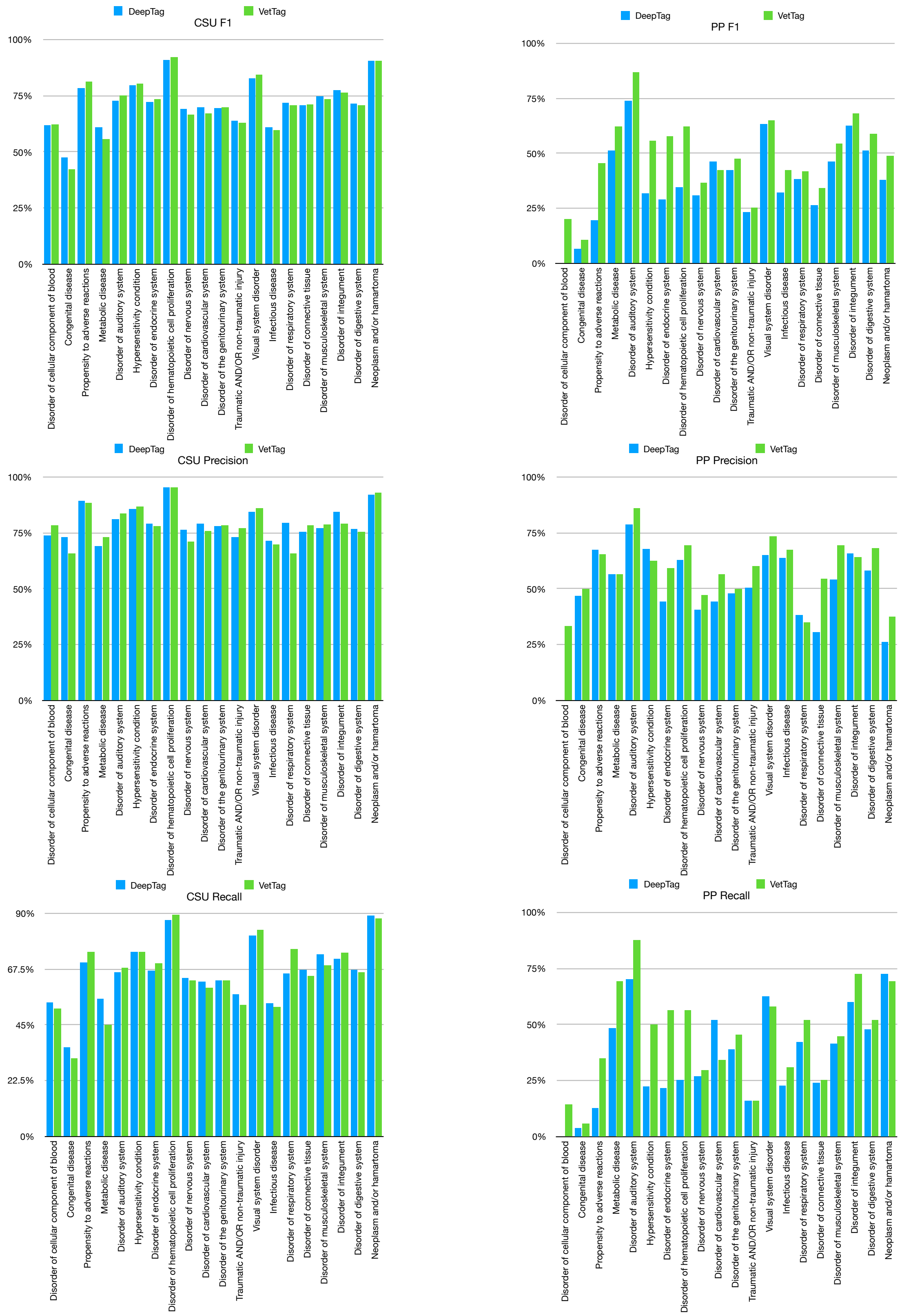

Supplementary Figure 3: DeepTag and VetTag comparison on the CSU and PP dataset for the 20 most frequent diagnosis categories.

Supplementary Tables

	CSU				PP			
	F_1	P	R	EM	F_1	P	R	EM
DeepTag	73.9	80.3	69.4	43.6	44.2	54.7	42.7	13.3
VetTag	73.6	79.8	69.1	49.3	53.8	61.2	51.0	23.2

Supplementary Table 1: Result comparison between DeepTag and VetTag on 41 top-level diagnosis categories.

Diagnosis	Words
Disorder of auditory system	ear, otitis, ears, therapy, yeast, allergy, assessment, infection, weeks, malassezia, allergic, dermatology, this, disease, medications, dermatitis, left, has, avalanche, not, topical, drops,
Disorder of immune function	eosinophilic, then, problem, todays, hypocalcemia, cornea, dose, skin, alt, weeks, prednisolone, not, ofloxacin, eosinophilia, old, rhinitis, duration, currently, medicine, cam, cephalexin, molly, pancytopenia, hyperglobulinemia, herpes,
Metabolic disease	diabetes, nph, hypercalcemia, glargine, vetsulin, weeks, home, insulin, amlodipine, dose, dehydration, culture, eye, last, visit, assessment, time, oncology, cll, vet, azotemia, units, ionized, lymphoma, carprofen, consistent, surgery,
Autoimmune disease	pemphigus, itp, lupus, mycophenolate, azathioprine, not, weeks, bear, dle, planum, diagnosed, due, assessment, thrombocytopenia, administration, tramadol, home, platelet, mediated,
Disorder of hematopoietic cell proliferation	lymphoma, multicentric, chop, doxorubicin, assessment, trial, continued, lsa, chemotherapy, cbc, lymph, protocol, oncology, diagnosed, treatment, home, well, ccnu, weeks, remission,
Neoplasm and/or hamartoma	oncology, lymphoma, osteosarcoma, sarcoma, mass, home, carcinoma, assessment, metastatic, adenocarcinoma, chemotherapy, multicentric, tumor, trial, has, surgery, disease, time, diagnosed, carboplatin, well, weeks, pulmonary, melanoma, treatment, metastasis, palladia,
Disorder of cardiovascular system	cardiology, hypertension, vasculitis, disease, current, home, at, assessment, valve, amlodipine, atenolol, infection, pimobendan, sildenafil, thrombus, pressure, heart, blood, weeks, not, arrhythmia, ventricular, pulmonary, internal, failure, echocardiogram, time, iliac, hours,
Infectious disease	pyoderma, assessment, infection, bacterial, therapy, uti, urinary, culture, superficial, this, dermatitis, treat, today, secondary, infections, well, problem, time, urine, upper, chloramphenicol, allergies, but, weeks, site, home,

Diagnosis	Words
Disorder of integument	assessment, otitis, therapy, pyoderma, mct, vinblastine, dermatology, weeks, trial, home, has, malassezia, ear, problem, metastatic, allergic, this, atopic, not, eyelid, medications, mass,
Traumatic AND/OR non-traumatic injury	fracture, wound, laceration, due, assessment, trauma, this, bandage, time, owner, fractured, eye, surgery, fractures, she, days, dog, may, joint, abrasion, home, radiographs, likely, change,
Disorder of cellular component of blood	thrombocytopenia, pancytopenia, itp, time, mycophenolate, count, azathioprine, prednisone, tramadol, dose, hemolytic, weeks, anemia, disease, leflunomide, steroids, eye, white, assessment, injury, future, problem, history, cbc,
Disorder of respiratory system	pneumonia, pulmonary, lung, nasal, epistaxis, adenocarcinoma, thoracocentesis, diagnosed, rhinitis, laryngeal, oncology, carcinoma, metastatic, paralysis, respiratory, assessment, home, mass, upper, revealed, necropsy, liver, consistent, chemotherapy, aspiration, srt, this, may, pneumothorax,
Vomiting	vomiting, ultrasound, chronic, assessment, findings, scan, skin, neoplasia, hematemesis, different, ddx, machine, nephrectomy, thickened, nodule, somewhat, ileum, not, intestines, last, bilateral,
Disorder of nervous system	laryngeal, seizures, his, meningioma, phenobarbital, seizure, home, signs, time, assessment, weeks, cytarabine, myelopathy, therapy, cricket, lesion, unremarkable, disease, hyperadrenocorticism, keppra, paralysis, tumor, neurology, levetiracetam, diagnosed, visit,
Hypersensitivity condition	dermatitis, allergic, therapy, atopic, otitis, pruritus, ears, assessment, allergies, dermatology, this, infection, weeks, treatment, not, ear, dvm, allergy, future, malassezia, time, today,
Anemia	pancytopenia, anemia, visit, hemolytic, persistent, steroids, hypertension, neoplasia, exam, thickening, calculi, white, inflammation, prednisolone, prednisone, treatments, vomiting, following, not,
Disorder of the genitourinary system	bladder, assessment, hematuria, tcc, urinary, urethra, mass, culture, uti, pyelonephritis, prostatic, cystitis, ureter, chemotherapy, diagnosed, testicle, therapy, piroxicam, disease, urine, not, prostate, revealed, carcinoma, renal, transitional, well, treatment, surgery,
Disorder of hemostatic system	thrombocytopenia, pancytopenia, itp, administration, prednisone, time, tramadol, bear, leflunomide, history, service, due, count, azathioprine, hypocalcemia, dose, mild, hypothyroidism, previous, steroids,
Propensity to	dermatitis, allergic, atopic, therapy, otitis,

Diagnosis	Words
adverse reactions	allergies, assessment, ears, infection, this, weeks, dermatology, pruritus, dvm, not, ear, trial, treatment, atopica, malassezia, atopy, today,
Poisoning	ingestion, assessment, toxicity, chocolate, vomiting, charcoal, not, maya, chance, activated, this, signs, dog, possible, rattlesnake, time, month, monitoring, therapy, marijuana,
Mental disorder	alopecia, screen, limb, issue,
Congenital disease	dysplasia, hip, bilateral, assessment, testicle, right, cerebellar, service, surgery, echo, congenital, options, buffalo, mild, signs, butternut, malformation, worse, reverse, pain, deformity, red, elbow, management,
Disorder of musculoskeletal system	osteosarcoma, assessment, osteoarthritis, surgery, dysplasia, ligament, left, disease, carboplatin, oncology, time, right, at, rupture, trial, diagnosed, fracture, amputation, this, joint, bilateral, cruciate, she, chemotherapy, tendon, lesion, home, weeks, presented, osa,
Disorder of endocrine system	methimazole, thyroid, weeks, levothyroxine, carcinoma, mass, hyperadrenocorticism, assessment, diabetes, diagnosed, home, disease, nph, trilostane, dose, time, may, hyperthyroidism, surgery, visit, glargine, eye,
Disorder of digestive system	dental, assessment, sac, adenocarcinoma, melanoma, mass, home, has, anal, time, oncology, carboplatin, anesthesia, left, metastatic, disease, this, enteropathy, necropsy, problem, not, surgery, oral, lip, liver, enteritis, from,
Visual system disorder	eye, ophthalmology, surgery, eyelid, assessment, sicca, time, uveitis, diagnosed, this, keratitis, cataract, treatment, mass, glaucoma, after, week, well, months, visit, infection,
Disorder of connective tissue	osteosarcoma, assessment, ligament, surgery, carboplatin, disease, dysplasia, rupture, cruciate, fracture, amputation, hip, weeks, right, diagnosed, left, trial, osa, chemotherapy, anesthesia, this, tendon, bilateral, oncology, joint, crcl, she, well,
Disorder of labor / delivery	level, progesterone, high, apparently, assessment, draw, healthy, days, puppies,
Disorder of pregnancy	progesterone, level, today, veterinary, measure, high, labor, pregnant, approximately, assessment, healthy, prior, once,

Supplementary Table 2: Most salient words in the model. Diagnosis categories without salient words are not shown.

Supplementary Materials

VetTag: improving automated veterinary diagnosis coding via large-scale language modeling

Yuhui Zhang[1,+], Allen Nie[2,+], Ashley Zehnder[2], Rodney L. Page[3], and James Zou[2,4,*]

[1]Department of Computer Science and Technology, Tsinghua University, Beijing, China
[2]Department of Biomedical Data Science, Stanford University, Stanford, CA 94305, USA
[3]Department of Clinical Sciences, Colorado State University, Fort Collins, CO, 80523, USA
[4]Chan-Zuckerberg Biohub, San Francisco, CA 94158, USA
[+]these authors contributed equally to this work
[*]Corresponding author. jamesz@stanford.edu

April 15, 2019

Contents

1 Dataset Details **6**

2 Model Details **7**
2.1 Text Encoder 7
2.2 Diagnosis Code Prediction 8

3 Experimental Setup **9**

4 Comparing VetTag and DeepTag **9**

5 Interpretation Details **9**

1 Dataset Details

We provide additional descriptive statistics of the dataset below. The training and evaluation CSU dataset, the external evaluation PP dataset and the unsupervised learning PSVG dataset are different due to the nature of the clinics. This additional information allows us to quantify the domain mismatch between CSU, PP and PSVG.

Length Distribution We plot a histogram to show the proportion of records in each dataset with the certain length in Supplementary Figure 1. Noticeably, CSU and PP follow the SOAP format ("Subjective, Objective, Assessment, Plan"), but PSVG data due to the API limitation, does not strictly follow this format. PSVG notes contain "History, Plan, Physical Exam" sections of the electronic medical record data. This causes the length of PSVG notes to be much shorter compared to CSU and PP notes.

Number of Labels Per Document Distribution We plot a histogram to show the proportion of records in each labeled dataset with the certain number of labels in Supplementary Figure 1. We do not have any labeled notes from PSVG and hence it is not included in the plot.

Species Distribution We plot pie charts to show the proportion of species in each labeled dataset in Supplementary Figure 2. CSU dataset contains a fair amount of notes across different species (e.g. equine, bovine, etc.), while PP being a suburban-based private clinic, the animal representations are much more focused on house pets.

2 Model Details

We formulate the problem of veterinary diagnosis coding as a multi-label classification problem. Given a veterinary record $\mathbf{X}$, which contains detailed description of the diagnosis, we try to infer a subset of diagnoses $\boldsymbol{y} \in \mathcal{Y}$, given a pre-defined set of diagnoses $\mathcal{Y}$. The problem of inferring a subset of diagnosis codes can be viewed as a series of independent binary prediction problems[1]. The binary classifier learns to predict whether a diagnosis code y_i exists or not for $i = 1, ..., m$, where $m = |\mathcal{Y}| = 4577$.

Our learning system has two components: a text encoder module and diagnosis code prediction module. In our work, we evaluated three text encoder modules: the convolutional neural network (CNN), the long short-term memory network (LSTM), which has demonstrated its effectiveness in learning implicit language patterns from the text[2], and the Transformer network, recently developed and proposed by Vaswani et al.[3]. Our diagnosis code prediction module consists of binary classifiers that are parameterized independently for each diagnosis.

2.1 Text Encoder

CNN The convolutional neural network (CNN) has been demonstrated to be effective for many NLP tasks[4]. Given a sequence of word embeddings $x_1, ..., x_T$, we apply a convolution operation with a window size of h (words) and a max-over-time pooling operation to get the summary vector c. The computation can be described in Eq 1, where $\oplus$ and $\otimes$ indicate the concatenation operator and the convolution operator, and tanh is the hyperbolic tangent function.

$$
\begin{aligned}
x_{1:T} &= x_1 \oplus x_2 \oplus ... \oplus x_T \\
c_i &= \tanh(w \otimes x_{i:i+h-1} + b) \\
\tilde{c} &= [c_1, c_2, ..., c_{T-h+1}] \\
c &= \max\{\tilde{c}\}
\end{aligned}
\tag{1}
$$

LSTM The long short-term memory network (LSTM) is a recurrent neural network with a long short-term memory cell[5]. A common LSTM network is composed of a hidden state h_t, a cell state c_t, an input gate i_t, an output gate o_t and a forget gate f_t. It maintains semantic gating functions specifically designed to capture long-term dependency between words. Given a sequence of word embeddings $x_1, ..., x_T$, the recurrent computation of LSTM network at a time step t can be described in Eq 2. σ is the sigmoid function: $\sigma(x) = 1/(1 + e^{-x})$, and $\odot$ indicates the Hadamard product.

$$
\begin{aligned}
f_t &= \sigma(W_f x_t + V_f h_{t-1} + b_f) \\
i_t &= \sigma(W_i x_t + V_i h_{t-1} + b_i) \\
o_t &= \sigma(W_o x_t + V_o h_{t-1} + b_o) \\
\tilde{c}_t &= \tanh(W_c x_t + V_c h_{t-1} + b_c) \\
c_t &= f_t \odot c_{t-1} + i_t \odot \tilde{c}_t \\
h_t &= o_t \odot \tanh(c_t)
\end{aligned}
\tag{2}
$$

Transformer The Transformer network was proposed by Vaswani et al. as a machine translation architecture[3]. We use a multi-layer Transformer setup similar to the one in Radford et al.[6]. The Transformer network is defined as a feed-forward network that starts at the word embedding level. Given the word embeddings of a sequence $x_1, ..., x_T \in \mathbb{R}^d$, we add positional embedding to such sequence so that the model can know the location of each word. We define this positional embedding $\text{PE} \in \mathbb{R}^{T \times d}$, where T is an arbitrarily set maximum length of a sequence (usually much longer than the longest sequence in our training dataset). For notation convenience, we let it equal to the sequence length T. However, since PE is generated as a cyclical sine-cosine wave and never updated during training, we can easily generate PE for sequence longer than T. For $i = 1, ..., d/2$, we can define the element in the PE matrix in Eq 3 (symbols inside parentheses indicate the coordinate of the element).

$$
\begin{aligned}
\text{PE}(t, 2i) &= \sin(t/10000^{2i/d}) \\
\text{PE}(t, 2i+1) &= \cos(t/10000^{2i/d})
\end{aligned}
\tag{3}
$$

We define the first input to the Transformer network: $H^0 = X + \text{PE}$, where $X = \{x_1, ..., x_T\}$ and PE is defined above. We note that $H^0 \in \mathbb{R}^{T \times d}$. Then for a given layer l, $l > 0$, we can define a feedforward *transformer block* in Eq 4. We let $W_k^{(i)}, W_q^{(i)}, W_v^{(i)}$ to have dimensions $(d/n) \times d$, and the resulting $H^{(i)}$ to have dimension $T \times (d/n)$. We additionally apply a mask M over the attention so that the model only looks at $< t$ steps when it generates the token at step t. For the same layer l, we repeat the above computation n times. This is referred as the multi-head attention computation, and n indicates the number of heads.

$$
\begin{aligned}
\begin{pmatrix} K^{(i)} \\ Q^{(i)} \\ V^{(i)} \end{pmatrix} &= \begin{pmatrix} W_k^{(i)} \\ W_q^{(i)} \\ W_v^{(i)} \end{pmatrix} H^{l-1} + \begin{pmatrix} b_k^{(i)} \\ b_q^{(i)} \\ b_v^{(i)} \end{pmatrix} \\
\tilde{H}^{(i)} &= V^{(i)}(\text{SoftMax}(\frac{Q^{(i)^T} K^{(i)}}{\sqrt{d}} \odot M)) \\
H^{(i)} &= W_h^{(i)} \tilde{H}^{(i)} + b_h^{(i)}
\end{aligned} \tag{4}
$$

After the multihead attention computation described above, we concatenate n $H^{(i)}$ matrices to obtain $\tilde{H}^l \in \mathbb{R}^{T \times d}$. We then apply a fully connected layer with ReLU activation function to this matrix and obtain the final hidden representation of the sequence for layer l: H^l. We describe the calculation in Eq 5. The matrix multiplication by $W_{o_1} \in \mathbb{R}^{D \times d}, W_{o_2} \in \mathbb{R}^{d \times D}$ are referred to as a bottleneck computation, where D is much larger than d. The Transformer network repeats the above computation to construct a multi-layer Transformer network.

$$
\begin{aligned}
\tilde{H}^l &= \text{Concat}(H^{(1)}, H^{(2)}, ..., H^{(n)}) \\
H^l &= W_{o_2} \text{ReLU}(W_{o_1} \tilde{H}^l + b_{o_1}) + b_{o_2}
\end{aligned} \tag{5}
$$

2.2 Diagnosis Code Prediction

We define a binary classifier for each of the 4577 diagnosis code in our pre-defined set. The binary classifier takes in a summary vector $\boldsymbol{c}$ that represents the veterinary record and outputs a sufficient statistic for the Bernoulli probability distribution indicating the probability of whether a diagnosis should be predicted (Eq 6).

$$
p(y_i) = \hat{y}_i = \sigma(w_i^T \boldsymbol{c} + b_i) \tag{6}
$$

Flat Training We use binary cross entropy loss averaged across all labels as the training loss for the flat training. Given the binary predictions from the model $\hat{\boldsymbol{y}} \in [0, 1]^m$ and correct binary label $\boldsymbol{y} \in \{0, 1\}^m$, binary cross entropy loss is written in Eq 7. The decision boundary in our model is set to be 0.5.

$$
\mathcal{L}_{\text{BCE}}(\hat{\boldsymbol{y}}, \boldsymbol{y}) = -\frac{1}{m} \sum_{i=1}^{m} y_i \log(\hat{y}_i) + (1 - y_i) \log(1 - \hat{y}_i) \tag{7}
$$

Hierarchical Training In this setting, we first define a adjacency matrix $\boldsymbol{M}$, where $\boldsymbol{M}_{ij} = 1$ if diagnostic code i is the child of diagnostic code j in the SNOMED-CT hierarchy, otherwise $\boldsymbol{M}_{ij} = 0$. during training time, we generate a mask $\boldsymbol{b} \in [0, 1]^m$. We generate the mask based on a recursive definition (Eq 8).

$$
b_i = \begin{cases} 1, y_j = 1 \text{ and } \boldsymbol{M}_{ij} = 1 \\ 0, \text{ otherwise} \end{cases} \tag{8}
$$

Then we can easily apply this mask to both $\boldsymbol{y}$ the ground truth label as well as the $\hat{\boldsymbol{y}}$. This masking vector allows us to only penalize the prediction of a diagnostic code when its parent is present, thus greatly reducing the number of negative examples for rare diagnoses. We compute the hierarchical binary cross-entropy loss in Eq 9.

$$
\mathcal{L}_{\text{H-BCE}}(\hat{\boldsymbol{y}}, \boldsymbol{y}) = -\frac{1}{\sum_{i=1}^{m} b_i} \sum_{i=1}^{m} [y_i \log(\hat{y}_i) + (1 - y_i) \log(1 - \hat{y}_i)] \cdot b_i \tag{9}
$$

During inference time, we generate masking vector $\hat{\boldsymbol{b}}$ by setting $\hat{b}_i = 1$ when all the ancestors of the diagnosis code i are predicted as true, and produce the final model prediction as $\hat{\boldsymbol{y}} \cdot \hat{\boldsymbol{b}}$.

3 Experimental Setup

We describe our experimental setup in the following section. We truncate all documents to no more than 600 tokens, padded with start and end of sentence tokens. This step is helpful in reducing computational requirement.

Neural Network Architecture In order to have a fair comparison among encoders—CNN, LSTM and Transformer—we set all the latent dimension as 768. For the CNN, we use 384 kernels for convolution with the kernel size of 4. For the LSTM, we compare the performance of unidirectional LSTM and bidirectional LSTM. For the Transformer, we stack 6 transformer blocks, with 8 heads for the multi-head attention on each layer. We let the feedforward dimension to be 2048.

Pretraining All pretraining is conducted on the PSVG dataset. We investigate the effect of pretraining the word embedding (+W) and pretraining the encoder with language modeling objective (+P). In the word embedding pretraining, we use the Word2Vec algorithm[7] on the PSVG dataset. The word embedding dimension is set to 768. For the pretraining language modeling objective, we initialize word embeddings with Xavier initialization[8] and directly optimize $-\log P(X)$.

Training We implement our model in PyTorch. We use Noam Optimizer[3] with 8000 warm up steps. The dropout rate is set to 0.1 during training to reduce overfitting. All models are trained for 10 epochs. We use a batch size of 5 for each model, which is the maximum allowed to train VetTag on a single GPU.

MetaMap Baseline We use the popular MetaMap, a program developed by the National Library of Medicine (NLM)[9], as a baseline. MetaMap processes a document and outputs a list of matched medically-relevant keywords with its frequencies in the given document. We use MetaMap as a feature extractor, mapping each document into a frequency-encoded bag-of-words vector. The final feature vector size is 57,235. We perform the multi-label classification task with SVM and MLP with feature vectors.

4 Comparing VetTag and DeepTag

DeepTag is designed to make predictions on the 42 top-level diagnosis categories[10]. We restrict the performance of VetTag to these top-level categories except for clinical finding (the spurious category) in order to directly compare its performance head-to-head with DeepTag. Note that VetTag is optimized not to predict just on these categories but on all 4577 categories; hence the comparison is more favorable for DeepTag. We report the result comparison in Supplementary Table 1. On the PP test data, VetTag substantially outperforms DeepTag for both F_1 and exact match (EM). On the CSU test data, VetTag achieved better EM score and comparable F_1 score as DeepTag. Supplementary Figure 3 provides the comparison of VetTag and DeepTag for the 20 most frequent categories, demonstrating the superior performance of VetTag.

5 Interpretation Details

We compute the standard saliency map for each input text; this is defined as the input vector multiplied by the gradient of the predicted probability with respect to the input. The saliency of each word quantifies the influence of that word on VetTag's predictions. For each of the 41 top-level diagnosis categories, we select the top 50 words that have the highest saliency for that diagnosis, defined as the words with saliency score ≥ 0.2 are the largest number of clinical notes labeled with the diagnosis. We then intersect the 50 most salient words with the MetaMap expert-curated dictionary in order to select the most medically relevant words. These words are shown in Supplementary Table 2.

References

1. Mohammad S Sorower. A literature survey on algorithms for multi-label learning. *Oregon State University, Corvallis*, 18, 2010.

2. Tomáš Mikolov. Statistical language models based on neural networks. *Presentation at Google, Mountain View, 2nd April*, 2012.

3. Ashish Vaswani et al. Attention is all you need. In *Advances in Neural Information Processing Systems*, pages 5998–6008, 2017.

4. Yoon Kim. Convolutional neural networks for sentence classification. *arXiv preprint arXiv:1408.5882*, 2014.

5. Sepp Hochreiter and Jürgen Schmidhuber. Long short-term memory. *Neural computation*, 9(8):1735–1780, 1997.

6. Alec Radford, Karthik Narasimhan, Tim Salimans, and Ilya Sutskever. Improving language understanding by generative pre-training. 2018.

7. Tomas Mikolov, Ilya Sutskever, Kai Chen, Greg S Corrado, and Jeff Dean. Distributed representations of words and phrases and their compositionality. In *Advances in neural information processing systems*, pages 3111–3119, 2013.

8. Xavier Glorot and Yoshua Bengio. Understanding the difficulty of training deep feedforward neural networks. In *Proceedings of the thirteenth international conference on artificial intelligence and statistics*, pages 249–256, 2010.

9. Alan R Aronson and François-Michel Lang. An overview of metamap: historical perspective and recent advances. *Journal of the American Medical Informatics Association*, 17(3):229–236, 2010.

10. Allen Nie et al. Deeptag: inferring diagnoses from veterinary clinical notes. *npj Digital Medicine*, 1(1):60, 2018.
